# Protective Effects of Nerolidol on Thrombotic Events, Systemic Inflammation, Oxidative Stress, and DNA Damage Following Pulmonary Exposure to Diesel Exhaust Particles

**DOI:** 10.3390/biomedicines13030729

**Published:** 2025-03-17

**Authors:** Naserddine Hamadi, Sumaya Beegam, Nur Elena Zaaba, Ozaz Elzaki, Alreem Alderei, Maha Alfalahi, Shamma Alhefeiti, Dana Alnaqbi, Salama Alshamsi, Abderrahim Nemmar

**Affiliations:** 1 Department of Life and Environmental Sciences, College of Natural and Health Sciences, Zayed University, Abu Dhabi P.O. Box 144534, United Arab Emirates; naserddine.hamadi@zu.ac.ae; 2 Department of Physiology, College of Medicine and Health Sciences, United Arab Emirates University, Al Ain P.O. Box 15551, United Arab Emirates; sumayab@uaeu.ac.ae (S.B.); elenazaaba@uaeu.ac.ae (N.E.Z.); ozazelzaki@uaeu.ac.ae (O.E.); 202119379@uaeu.ac.ae (A.A.); 202113435@uaeu.ac.ae (M.A.); 202003883@uaeu.ac.ae (S.A.); 202111316@uaeu.ac.ae (D.A.); 202115812@uaeu.ac.ae (S.A.); 3 Zayed Center for Health Sciences, United Arab Emirates University, Al Ain P.O. Box 15551, United Arab Emirates

**Keywords:** nerolidol, diesel exhaust particles, platelet aggregation, endothelial dysfunction, oxidative stress, DNA damage

## Abstract

**Background/Objectives**: Inhalation of environmental particulate air pollution has been reported to cause pulmonary and systemic events including coagulation disturbances, systemic inflammation, and oxidative stress. Nerolidol, a naturally occurring sesquiterpene alcohol, has effective antioxidant and anti-inflammatory effects. Hence, the aim in the present investigation was to evaluate the potential ameliorative effects of nerolidol on the coagulation and systemic actions induced by pulmonary exposure to diesel exhaust particles (DEPs). **Methods**: Nerolidol (100 mg/kg) was given to mice by oral gavage one hour before the intratracheal instillation of DEPs (0.5 mg/kg), and 24 h later various markers of coagulation and systemic toxicity were evaluated. **Results**: Nerolidol treatment significantly abrogated DEP-induced platelet aggregation in vivo and in vitro. Nerolidol has also prevented the shortening of the prothrombin time and activated plasma thromboplastin time triggered by DEP exposure. Likewise, while the concentrations of fibrinogen and plasminogen activator inhibitor-1 were increased by DEP administration, that of tissue plasminogen activator was significantly decreased. These effects were abolished in the group of mice concomitantly treated with nerolidol and DEP. Moreover, plasma markers of inflammation, oxidative stress, and endothelial dysfunction which were significantly increased in the DEP-treated group, returned to control levels in the nerolidol + DEP group. Nerolidol treatment significantly ameliorated the increase in the concentrations of hypoxia-inducible factor 1α, galectin-3, and neutrophil gelatinase-associated lipocalin induced by pulmonary exposure to DEP. The co-administration of nerolidol + DEPs significantly mitigated the increase in markers of oxidative DNA damage, 8-hydroxy-2-deoxyguanosine, and apoptosis, cleaved-caspase-3, induced by DEP. **Conclusions**: Collectively, our data demonstrate that nerolidol exert significant ameliorative actions against DEP-induced thrombotic events, endothelial dysfunction, systemic inflammation, oxidative stress, DNA damage, and apoptosis. Pending further pharmacological and toxicological studies, nerolidol could be a promising agent to alleviate the toxicity of inhaled DEPs and other pollutant particles.

## 1. Introduction

Dietary phytochemicals are naturally occurring compounds synthesized by plants that exhibit various beneficial health effects [1,2,3]. These bioactive compounds can be broadly categorized into phenolic compounds (such as flavonoids and tannins), glucosinolates, alkaloids, and terpenoids [1,2,3]. Once consumed, phytochemicals participate in numerous biological processes within the human body, including redox reactions, cell signaling, modulation of inflammation, and oxidative stress [1,2,3]. Currently, natural products are gaining attention as a promising source for the treatment of various inflammatory conditions [1,2,3].

Ambient air pollution consists of a complex mixture of various chemical substances, including solid and liquid particles, hydrocarbons, metals, and gases. Among the various pollutants, particulate matter (PM) is most strongly linked to cardiovascular events [4,5,6]. PM is classified by particle size: PM10 refers to particles with a diameter of less than 10 µm, PM2.5 to those under 2.5 µm, and ultrafine PM0.1, also known as nanoparticles, measures less than 100 nm [7]. Diesel engine exhaust is a major source of PM2.5 in urban environments and a primary contributor to combustion-derived nanoparticles [8]. Due to this, diesel exhaust is frequently used as a representative model for particulate air pollution in experimental research studies [8,9]. Clinical and experimental studies have reported that pulmonary exposure to DEPs can adversely influence critical cardiovascular parameters, including alterations in blood pressure, heart rate variability, endothelial dysfunction, and an increased propensity for thrombosis through mechanisms involving inflammation and oxidative injury [10,11].

Given the substantial evidence that DEP-induced thrombotic and systemic toxicity is largely driven by oxidative stress and inflammation, there is a strong rationale for exploring targeted interventions to mitigate DEP exposure and its associated toxicity by using safe phytochemical agents with potent antioxidant and anti-inflammatory properties. While nerolidol is well-known for its robust antioxidant and anti-inflammatory effects, its specific role in mitigating DEP-induced systemic and coagulatory disturbances remains unexplored.

Nerolidol, also known as 3,7,11-trimethyl-1,6,10-dodecatrien-3-ol, is an aliphatic sesquiterpene alcohol commonly found in the essential oils of various fragrant flowers and plants. It is characterized by its distinct floral aroma and can be found in a wide range of sources, including neroli, citronella, lemongrass, ginger, rose, and tea tree [12,13]. Of note, the United States Food and Drug Administration has categorized nerolidol as generally recognized as safe (GRAS) and has approved its use as a flavor enhancer in food industry [13]. Nerolidol has been shown to exert a protective effect against isoproterenol-induced acute myocardial infarction and to ameliorate hypertension-induced cardiac remodeling in rats [14,15]. Moreover, nerolidol has been reported to inhibit lipopolysaccharide-induced acute lung injury by preventing alveolar-capillary barrier disruption, lung edema, and lipid peroxidation through the modulation of antioxidant enzymes [16]. Also, nerolidol supplementation has been found to exert a neuroprotective action against neuroinflammation and oxidative stress induced by rotenone-induced experimental model of Parkinson’s disease [17].

Therefore, our study aims to investigate the potential ameliorative effects of nerolidol on coagulatory and systemic disturbances resulting from pulmonary exposure to DEP, and the underlying mechanisms of these effects. To this end, various biomarkers and endpoints were assessed in this work, including peripheral thrombosis, endothelial dysfunction, systemic inflammation, oxidative stress, DNA damage, and apoptosis.

## 2. Materials and Methods

### 2.1. Particles and Nerolidol

The diesel exhaust particles (DEPs) were obtained from the National Institute of Standards and Technology in Gaithersburg, MD, USA. These particles were suspended in sterile saline (0.9%) with the addition of 0.01% Tween 80. Before being diluted and administered intratracheally (i.t.), the DEP suspensions underwent continuous sonication for 15 min in a Clifton Ultrasonic Bath (Clifton, NJ, USA), followed by vortexing. Control mice were given an intratracheal (i.t.) instillation of saline with 0.01% Tween 80. A previous transmission electron microscopy examination of the DEP used in this study revealed the presence of small carbonaceous particle aggregates measuring under 0.1 µm, along with larger aggregates with diameters less than 1 µm [18]. Additionally, nerolidol was purchased from Sigma Chemical (St. Louis, MO, USA).

### 2.2. Animals and Treatments

BALB/C mice, around 8 weeks old and weighing approximately 20 g, were kept in controlled environments with a 12 h light/12 h dark cycle at 23 ± 2 °C. They had free access to tap water and standard commercial chow (National Feed and Flour and Marketing Co., Abu Dhabi, United Arab Emirates).

To deliver DEPs into the lungs, i.t. instillation was used [19,20]. The latter technique is a relevant method for pulmonary exposure in experimental studies involving rodents because it allows precise control over the dose, timing, and localization of substances delivered directly to the lungs [19,21]. Mice were first anesthetized with isoflurane (5%) using a Surgivet^®^ model 100 vaporizer. They were then positioned supine with extended necks on an angled board, and a 24-gauge Becton Dickinson cannula was carefully introduced through the mouth into the trachea. A suspension of DEPs at a dose of 0.5 mg/kg or a saline solution (0.9% NaCl with 0.01% Tween 80) was instilled into the trachea using a sterile syringe in a volume of 100 µL, immediately followed by a 100 µL air bolus.

Nerolidol was administered orally by gavage at a dose of 100 mg/kg one hour prior to the i.t. instillation of either DEPs or saline. The oral administration of nerolidol 1 h prior to DEP exposure is based on previous studies that adopted similar preventive approach in acute studies [11,16,17,22]. This dose was selected based on previous research that demonstrated its safety and efficacy across various animal models, such as traumatic brain injury, thioacetamide-induced oxidative damage in the kidney, colon inflammation, and isoproterenol-induced acute myocardial infarction [14,23,24,25].

The mice were randomly divided into four groups with equal numbers (*n* = 8), receiving the following treatments:

- Group 1: Received normal saline given by oral gavage one hour before the i.t. instillation of saline.
- Group 2: Received normal saline given by oral gavage one hour before i.t. instillation of DEPs (0.5 mg/kg).
- Group 3: Received nerolidol given by oral gavage (100 mg/kg) one hour before the i.t. instillation of saline.
- Group 4: Received nerolidol given by gavage (100 mg/kg) one hour before i.t. instillation of DEPs (0.5 mg/kg).

**Figure 1:**
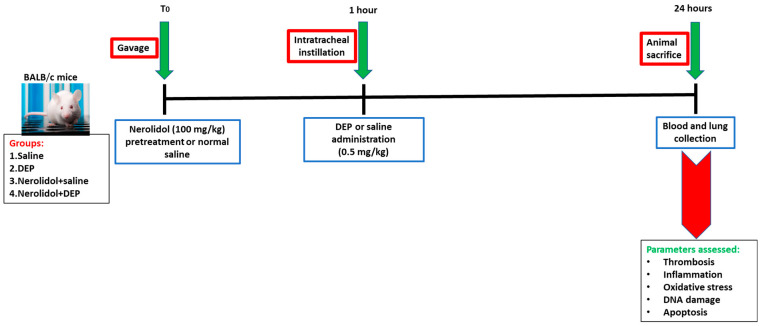
Flow chart displaying treatment and endpoints measured. Nerolidol (100 mg/kg) or normal saline was first given by gavage to BALB/c mice, then one hour later either saline or diesel exhaust particles (DEP; 0.5 mg/kg) were administered by intratracheal instillation. DEPs were suspended in sterile saline (0.9%) with the addition of 0.01% Tween 80, sonicated continuously for 15 min using a Clifton Ultrasonic Bath followed by vortexing. Saline solution (0.9% NaCl with 0.01% Tween 80) was given to the control group.

Twenty-four hours after the administration of DEPs or saline into the lungs, various parameters were evaluated (Figure 1).

#### 2.2.1. Pial Microvessels Thrombosis Model

To evaluate in vivo thrombosis, experiments were conducted on mice given either DEPs or saline, with or without nerolidol. The study focused on pial arterioles and venules, employing a previously established method for thrombosis assessment in these microvessels [11,26,27]. Briefly, mice were anesthetized with urethane (1 mg/g body weight, intraperitoneally). Following intubation of the trachea, a 2F venous catheter (Portex, Hythe, UK) was inserted into the right jugular vein for fluorescein administration (Sigma-Aldrich, St. Louis, MO, USA). A craniotomy was then performed on the right temporoparietal cortex using a hand-held microdrill, and the dura mater was carefully retracted. Only preparations without signs of trauma were used; any showing microvascular or underlying brain damage were excluded. Cerebral microcirculation was observed directly using a fluorescence microscope (Olympus, Melville, NY, USA) attached to a camera and DVD recorder. The body temperature was maintained at 37 °C using a heating pad and monitored via a rectal thermoprobe connected to a temperature reader (Physitemp Instruments, Clifton, NJ, USA). A field containing arterioles and venules with diameters between 15 and 20 μm was selected and recorded before and during the photochemical injury. The injury was induced by administering fluorescein (0.1 mL of a 5% solution per mouse) through the jugular vein, allowing it to circulate for 30–40 s. The cranial area was then exposed to stabilized mercury light, triggering platelet adhesion and aggregation at the endothelial injury site. This led to the progressive growth of platelet aggregates and thrombus formation until complete vascular occlusion occurred. The time from injury to complete occlusion (flow stop) in both arterioles and venules was recorded in seconds. At the end of the experiment, the animals were euthanized with a lethal overdose of urethane.

#### 2.2.2. Whole Blood Platelet Aggregation Ex Vivo

Ex vivo platelet aggregation in whole blood was evaluated using a formerly established method [11,26,27]. Mice were first exposed to either DEPs or saline, with or without nerolidol administration. They were then anesthetized through an intraperitoneal injection of sodium pentobarbital (45 mg/kg), and blood samples were drawn from the inferior vena cava into a solution containing 3.8% citrate as an anticoagulant. A 100 µL portion of each blood sample was placed in a coagulometer (MC 1 VET, Merlin, Lemgo, Germany) for analysis. The samples were incubated for 3 min with 0.1 µM adenosine diphosphate (ADP) and subsequently stirred for an additional 3 min. After this process, 25 µL of the blood was removed and immediately stabilized on ice using CellFix solution (225 mL) from Becton Dickinson (Franklin Lakes, NJ, USA). The next step involved counting individual platelets with a VET ABX Micros system equipped with a specialized mouse card (Montpellier, France). The extent of platelet aggregation was then determined by measuring the reduction in the number of single platelets, which indicated ADP-induced aggregation at a concentration of 0.1 µM [11,26,27]. In the present study, DEPs and nerolidol were not tested in the absence of ADP in platelet aggregation experiment. The blood samples were collected from mice exposed to either DEPs or saline, with or without nerolidol administration, and incubated with 0.1 µM ADP. The latter is a platelet agonist that promotes platelet aggregation [28]. The purpose of this approach was to assess whether the platelet pro-aggregatory effect of ADP is potentiated by the in vivo exposure to DEPs and to determine whether the presence of nerolidol influences this effect [11,26,27].

#### 2.2.3. The In Vitro Measurement of Activated Partial Thromboplastin Time (aPTT) and Prothrombin Time (PT)

The measurement of PT and aPTT was conducted using previously established protocols [29,30]. After the i.t. administration of either DEPs or saline directly into the lungs of the mice, with or without nerolidol treatment, the animals were anesthetized as previously described. Blood samples were drawn from the inferior vena cava and mixed with a 3.8% citrate solution at a 9:1 ratio of blood to anticoagulant. Platelet-poor plasma was then isolated from the blood, and PT was measured using recombiplastin, a human recombinant relipidated thromboplastin, provided by Instrumentation Laboratory (Orangeburg, NY, USA) and analyzed using a coagulometer (MC 1 VET, Merlin, Lemgo, Germany). Likewise, the aPTT was evaluated with an automated aPTT reagent (bioMerieux, Durham, NC, USA) using the same coagulometer, providing a precise assessment of both clotting times.

#### 2.2.4. Blood Collection for Biochemical Analysis

Following exposure to either saline or DEP, with or without the administration of nerolidol, the animals were anesthetized according to the previously established procedure. Blood samples were drawn from the inferior vena cava and collected in tubes containing 4% EDTA to prevent clotting. The blood was then centrifuged at 900× *g* for 15 min at a temperature of 4 °C to separate the plasma. The removed plasma samples were stored at −80 °C until further biochemical evaluations could be conducted.

The quantification of the concentrations of fibrinogen (Molecular Innovation, Southfield, MI, USA), tissue plasminogen-activator (tPA; Molecular Innovation, Southfield, MI, USA) and plasminogen activator inhibitor-1 (PAI-1; Molecular Innovation, Southfield, MI, USA), C-reactive protein (CRP; Uscn Life Science Inc., Wuhan, China), and interleukin (IL)-1β (R & D systems, Minneapolis, MN, USA) were measured using commercially available ELISA kits.

The catalase activity was measured with a colorimetric activity assay kit purchased from Cayman Chemical (Ann Arbor, MI, USA). Reactive oxygen species (ROS) were quantified using 2′,7′-Dichlorofluorescein diacetate (DCFDA; Molecular Probes, Eugene, OR, USA) as a fluorescent indicator, following previously established protocols [31,32]. Nitric oxide (NO) levels were determined using a total NO assay kit from R&D Systems (Minneapolis, MN, USA), which measures the more stable NO metabolites NO_2_^−^ and NO_3_^−^ [33,34].

The measurement of the plasma concentration of adhesion molecules including E-selectin, P-selectin, intercellular adhesion molecule-1 (ICAM-1), and vascular cell adhesion molecule-1 (VCAM-1) was carried out with commercially available ELISA kits procured from R&D systems (Duo Set, Minneapolis, MN, USA).

The concentrations of hypoxia-inducible factor 1α (HIF-1α), galectin-3, and neutrophil gelatinase-associated lipocalin (NGAL) were assayed by means of commercially available ELISA kits from R&D systems (Duo Set, Minneapolis, MN, USA).

The levels of cleaved caspase-3 (R&D Systems (Minneapolis, MN, USA) and 8-hydroxy-2′-deoxyguanosine (8-OH-dG; Cayman Chemicals, Ann Arbor, MI, USA) were measured utilizing kits, following the detailed protocol provided by the respective manufacturer.

### 2.3. Statistical Analysis

Statistical analysis was conducted using version 7 of GraphPad Prism (GraphPad Software Inc., San Diego, CA, USA). Group comparisons were made using one-way analysis of variance (ANOVA) followed by Holm–Sidak’s multiple comparisons test. *p*-values less than 0.05 were considered statistically significant.

## 3. Results

### 3.1. In Vivo Thrombosis in Pial Microvessels

**Figure 2:**
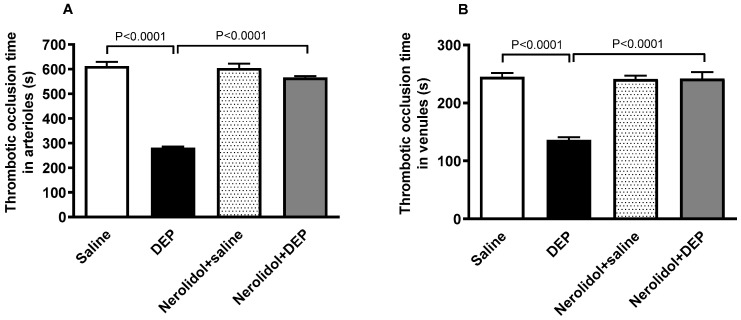
Thrombotic occlusion times in pial arterioles (**A**) and venules (**B**) assessed 24 h after intratracheal instillation of either saline or diesel exhaust particles (DEP; 0.5 mg/kg), with or without pretreatment with nerolidol (100 mg/kg) administered one hour prior. Data are means ± SEM (*n* = 8). Statistical analysis was performed using one-way analysis of variance with subsequent application of Holm–Sidak’s multiple comparisons test.

The i.t. instillation of DEPs led to a marked increase in thrombotic events within the pial microvessels, as evidenced by significantly reduced thrombotic occlusion times (*p* < 0.0001) (Figure 2A,B). In contrast, in pial arterioles, the group treated with nerolidol prior to DEP administration demonstrated a significant improvement, showing prolonged occlusion times compared with the DEP-only group (*p* < 0.0001) (Figure 2A). Likewise, in the pial venules, the nerolidol treatment prevented the shortening of thrombotic occlusion time induced by DEP exposure (*p* < 0.0001) (Figure 2B).

### 3.2. Ex Vivo Platelet Aggregation in Whole Blood

**Figure 3:**
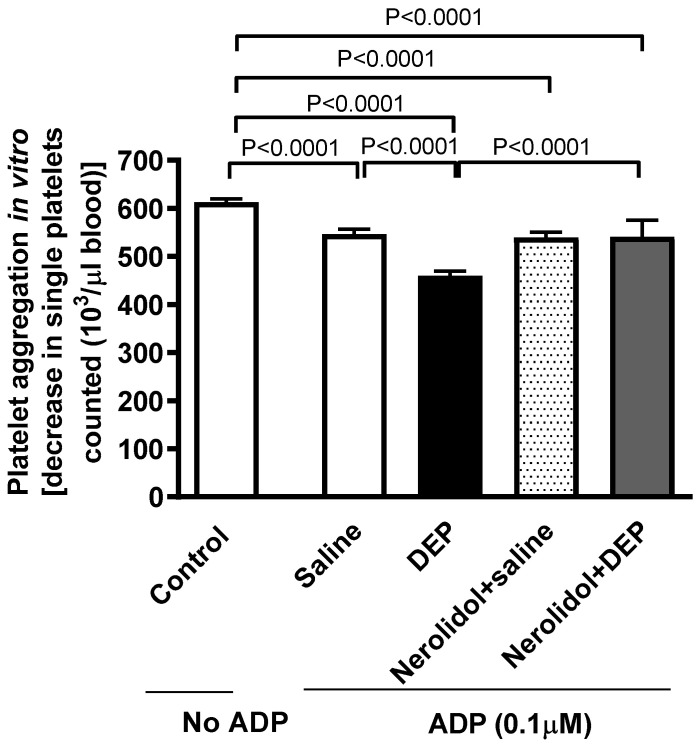
Platelet aggregation in whole blood incubated with ADP (0.1 µM). The blood was collected from mice, 24 h after intratracheal instillation of either saline or diesel exhaust particles (DEP; 0.5 mg/kg), with or without pretreatment with nerolidol (100 mg/kg) administered one hour prior. Platelet aggregation was evaluated by assessing the drop in single platelets, counted as a result of aggregation induced by DEPs. Data are means ± SEM (*n* = 8). Statistical analysis was performed using one-way analysis of variance with subsequent application of Holm–Sidak’s multiple comparisons test.

Whole blood collected from mice given i.t. instillation of DEPs showed an enhanced platelet aggregation in response to incubation with ADP (0.1 µM), resulting in a significant reduction in the single platelet count (*p* < 0.0001) (Figure 3). Treatment of mice with nerolidol by gavage markedly alleviated DEP-induced platelet aggregation (*p* < 0.0001) (Figure 3).

### 3.3. Activated Partial Thromboplastin Time and Prothrombin Time

**Figure 4:**
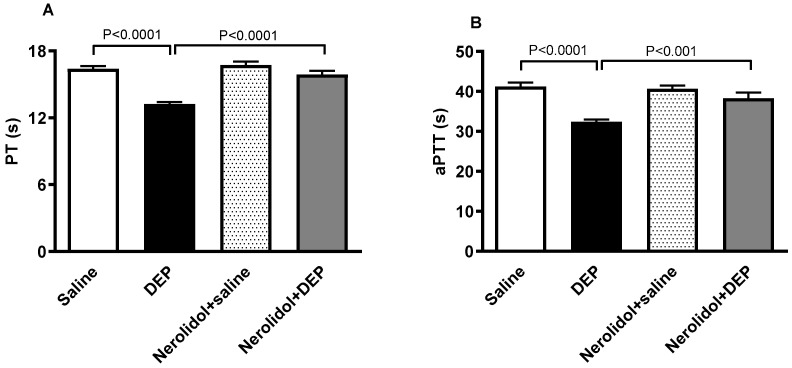
The prothrombin time (PT; (**A**)) and activated partial thromboplastin time (aPTT; (**B**)) assessed on plasma samples, assessed 24 h after intratracheal instillation of either saline or diesel exhaust particles (DEP; 0.5 mg/kg), with or without pretreatment with nerolidol (100 mg/kg) administered one hour prior. Data are means ± SEM (*n* = 8). Statistical analysis was performed using one-way analysis of variance with subsequent application of Holm–Sidak’s multiple comparisons test.

The effect of DEPs or saline with or without nerolidol treatment on PT and aPTT is shown in Figure 4. DEPs induced a significant shortening of PT (*p* < 0.0001; Figure 4A), an effect that was significantly prevented by nerolidol pretreatment (*p* < 0.0001; Figure 4A). Similarly, the aPTT was significantly reduced by i.t. instillation of DEPs (*p* < 0.0001; Figure 4B). This effect was abolished in the group of mice concomitantly treated with nerolidol and DEPs (*p* < 0.001; Figure 4B).

### 3.4. Markers of Coagulation and Fibrinolysis

**Figure 5:**
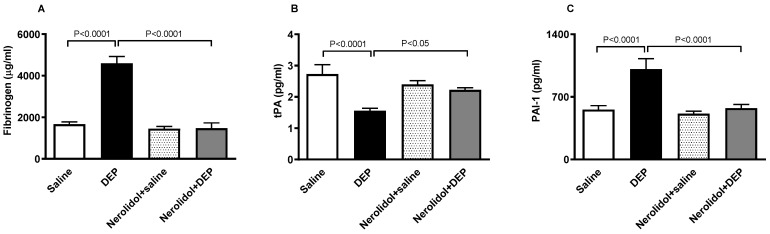
Fibrinogen (**A**), tissue plasminogen activator (tPA; (**B**)), and plasminogen activator inhibitor-1 (PAI-1; (**C**)) concentrations in the plasma, assessed 24 h after intratracheal instillation of either saline or diesel exhaust particles (DEP; 0.5 mg/kg), with or without pretreatment with nerolidol (100 mg/kg) administered one hour prior. Data are means ± SEM (*n* = 8). Statistical analysis was performed using one-way analysis of variance with subsequent application of Holm–Sidak’s multiple comparisons test.

Figure 5A shows that compared with the control group, pulmonary exposure to DEPs induced a substantial augmentation of the concentration of fibrinogen in the plasma (*p* < 0.0001). This effect was abolished in the group of mice pretreated with nerolidol and exposed to DEPs (*p* < 0.0001). On the other hand, Figure 5B illustrates that the i.t. instillation of DEPs caused a significant decrease in the concentration of tPA in the plasma (*p* < 0.0001), and that this effect has been prevented in mice concomitantly treated with nerolidol and DEPs (*p* < 0.05). Likewise, as displayed in Figure 5C, the increase in the concentration of PAI-1 triggered by DEPs (*p* < 0.0001) has been abrogated in nerolidol + the DEP group (*p* < 0.0001).

### 3.5. Markers of Inflammation and Oxidative Stress

**Figure 6:**
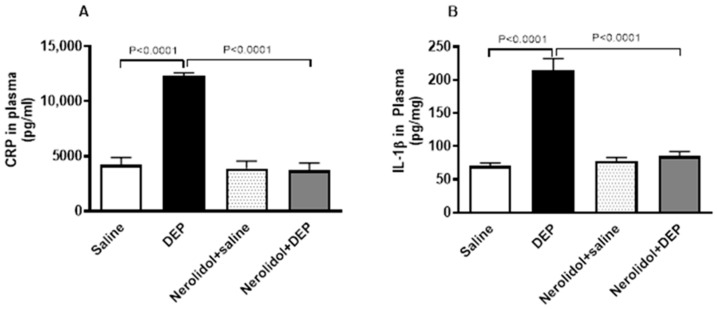
C-reactive protein (CRP; (**A**)) and interleukin-1β (**B**), assessed 24 h after intratracheal instillation of either saline or diesel exhaust particles (DEP; 0.5 mg/kg), with or without pretreatment with nerolidol (100 mg/kg) administered one hour prior. Data are means ± SEM (*n* = 8). Statistical analysis was performed using one-way analysis of variance with subsequent application of Holm–Sidak’s multiple comparisons test.

Compared with saline-treated mice, pulmonary exposure to DEPs noticeably increased the concentrations of CRP (*p* < 0.0001) and IL-1β (*p* < 0.0001) (Figure 6). However, nerolidol pretreatment effectively prevented the increase in markers of inflammation induced by DEPs (*p* < 0.0001).

### 3.6. Markers of Oxidative Stress

**Figure 7:**
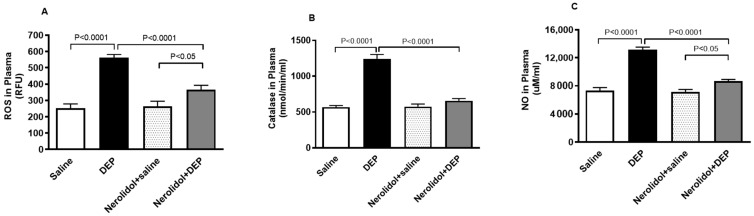
Reactive oxygen species (ROS; (**A**)), catalase (**B**), and nitric oxide (NO; (**C**)) levels in the plasma, assessed 24 h after intratracheal instillation of either saline or diesel exhaust particles (DEP; 0.5 mg/kg), with or without pretreatment with nerolidol (100 mg/kg) administered one hour prior. Data are means ± SEM (*n* = 8). Statistical analysis was performed using one-way analysis of variance with subsequent application of Holm–Sidak’s multiple comparisons test.

Figure 7A demonstrates that pulmonary exposure to DEPs significantly increased plasma ROS levels compared with the control group (*p* < 0.0001). However, this increase was completely reversed in mice pretreated with nerolidol before DEP exposure (*p* < 0.0001). Similarly, Figure 7B shows that intratracheal instillation of DEPs led to a marked rise in plasma catalase activity (*p* < 0.0001), an effect that was prevented in mice treated with both nerolidol and DEPs (*p* < 0.0001). Furthermore, Figure 7C reveals that DEP exposure caused a substantial elevation in plasma NO levels (*p* < 0.0001), which was also abolished in the group receiving nerolidol and DEPs (*p* < 0.0001). There was a statistically significant increase in the levels of ROS (*p* < 0.05) and NO (*p* < 0.05) in DEPs + nerolidol vs. saline + nerolidol.

### 3.7. Markers of Vascular Dysfunction

**Figure 8:**
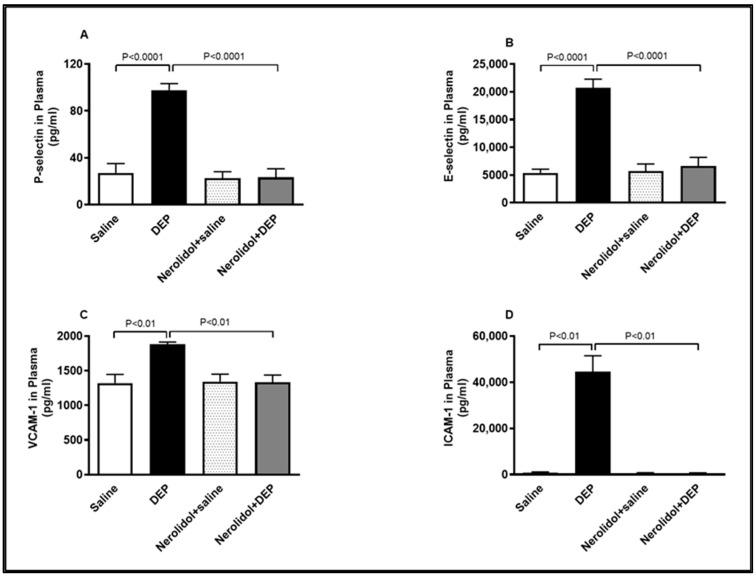
P-selectin (**A**), E-selectin (**B**), vascular cell adhesion molecule 1 (VCAM-1; (**C**)) and intercellular adhesion molecule-1 (ICAM-1; (**D**)) concentrations in the plasma, assessed 24 h after intratracheal instillation of either saline or diesel exhaust particles (DEP; 0.5 mg/kg), with or without pretreatment with nerolidol (100 mg/kg) administered one hour prior. Data are means ± SEM (*n* = 8). Statistical analysis was performed using one-way analysis of variance with subsequent application of Holm–Sidak’s multiple comparisons test.

Compared with the control group, intratracheal (i.t.) instillation of DEPs significantly increased plasma concentrations of P-selectin (*p* < 0.0001), E-selectin (*p* < 0.0001), VCAM-1 (*p* < 0.01), and ICAM-1 (*p* < 0.01) (Figure 8). However, the combination of nerolidol administered by gavage and pulmonary DEP exposure effectively prevented the rise in these markers of endothelial dysfunction (*p* < 0.0001–*p* < 0.01).

### 3.8. HIF-1α, Galectin-3 and NGAL Concentrations in the Plasma

**Figure 9:**
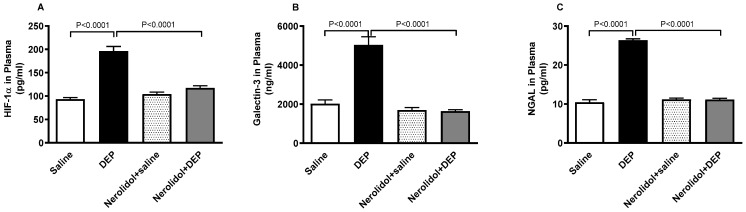
Hypoxia-inducible factor 1α (HIF-1α; (**A**)), galectin-3 (**B**) and neutrophil gelatinase-associated lipocalin (NGAL; (**C**)) concentrations in the plasma, assessed 24 h after intratracheal instillation of either saline or diesel exhaust particles (DEP; 0.5 mg/kg), with or without pretreatment with nerolidol (100 mg/kg) administered one hour prior. Data are means ± SEM (*n* = 8). Statistical analysis was performed using one-way analysis of variance with subsequent application of Holm–Sidak’s multiple comparisons test.

As shown in Figure 9, exposure to DEPs caused a substantial increase in HIF-1α (*p* < 0.0001), galectin-3 (*p* < 0.0001), and NGAL (*p* < 0.0001) concentrations in the plasma. However, co-treatment with nerolidol effectively counteracted the effects of DEPs, and successfully restored the concentrations of HIF-1α (*p* < 0.0001), galectin-3 (*p* < 0.0001), and NGAL (*p* < 0.0001) to levels comparable to the control group.

### 3.9. Markers of Apoptosis and Oxidative DNA Damage

**Figure 10:**
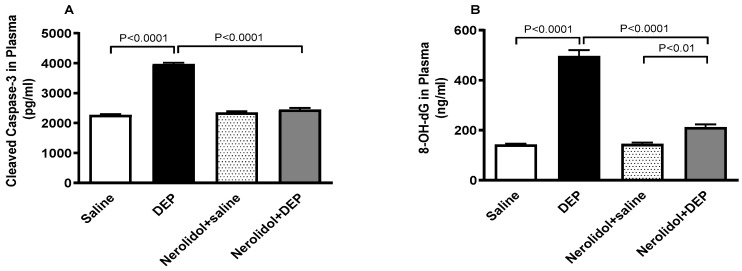
Cleaved caspase-3 (**A**) and 8-hydroxy-2′-deoxyguanosine (8-OH-dG; (**B**)) levels in the plasma, assessed 24 h after intratracheal instillation of either saline or diesel exhaust particles (DEP; 0.5 mg/kg), with or without pretreatment with nerolidol (100 mg/kg) administered one hour prior. Data are means ± SEM (*n* = 8). Statistical analysis was performed using one-way analysis of variance with subsequent application of Holm–Sidak’s multiple comparisons test.

Figure 10 shows that compared with control group, DEP exposure significantly increased the plasma levels of cleaved caspase-3 (*p* < 0.0001) and 8-OH-dG (*p* < 0.0001). Combining nerolidol with DEPs significantly alleviated the increase in caspase-3 (*p* < 0.0001) and 8-OH-dG (*p* < 0.0001) levels. There was a statistically significant increase in the levels of 8-OH-dG in DEPs + nerolidol vs. saline + nerolidol (*p* < 0.01).

## 4. Discussion

This study provides compelling evidence that nerolidol exhibits protective effects against DEP-induced vascular thrombosis, endothelial dysfunction, systemic inflammation, oxidative stress, DNA damage, and apoptosis. Additionally, nerolidol effectively counteracted the impact of DEP, restoring plasma concentrations of HIF-1α, galectin-3, and NGAL to control levels.

Although numerous experimental studies have demonstrated various effects of nerolidol—including anti-microbial, anti-parasitic, anti-biofilm, antioxidant, anti-nociceptive, anti-inflammatory, anti-ulcer, skin penetration enhancer, insect repellent, and anti-cancer properties [13] A thorough literature search reveals that information on the pharmacokinetics nerolidol remains scarce. However, two studies provide insight into this aspect. The first study, using a GC-MS method to analyze cis-/trans-nerolidol, examined the effects of a single oral dose of 1000 mg/kg in mice. The results showed that the maximum plasma concentration reached 0.27 μg/mL within 30 min and 0.35 μg/mL after 6 h, declining to near zero after 12 h. This study provides the only available report in the literature, with no subsequent studies to replicate the findings. Moreover, the sample size used was limited to just three mice [35]. Another study, utilizing LC-MS, investigated the pharmacokinetics of a 25 mg/kg intraperitoneal injection of nerolidol in rats. This study reported a peak plasma concentration of 8.30 μg/mL within 20 min, which declined to near zero after 2 h [36].

Ambient air pollution consists of a complex mixture of various chemical substances, including solid and liquid particles, hydrocarbons, metals, and gases. Among the various pollutants, particulate matter (PM) is most strongly linked to cardiovascular events [4,5,6]. PM is classified by particle size: PM10 refers to particles with a diameter of less than 10 µm, PM2.5 to those under 2.5 µm, and ultrafine PM0.1, also known as nanoparticles, measures less than 100 nm [7]. Diesel engine exhaust is a major source of PM2.5 in urban environments and a primary contributor to combustion-derived nanoparticles [8]. While diesel vehicles are gradually being phased out in developed countries, they remain prevalent in many developing countries. We do not know if nerolidol exerts similar beneficial effects on other forms of air pollution. Due to this, diesel exhaust is frequently used as a representative model for particulate air pollution in experimental research studies [8,9].

Clinical and experimental studies have reported that short-term exposure (lag 1-day exposure) to PM_2.5_ was significantly linked with cardiovascular complications [8,37,38,39,40]. Hence, in the present study, the acute effects (24 h) of a relevant type of particulate air pollution vs. DEPs have been assessed on vascular homeostasis. The dose of DEPs used in this study aligns with those commonly used in animal models including ours investigating the pathophysiological effects of particulate air pollution [22,41,42,43,44]. While this dose is higher than typical human exposure in traffic over a few hours, it is intended to replicate the effects of short-term, high air pollution episodes, particularly those involving particulate matter ≤10 μm (PM10). However, one must consider that, in comparison with PM10, DEPs comprise a substantial amount of ultrafine particles which are known to have higher surface area and particle numbers.

Exposure to inhaled particulate air pollution is linked to both acute events, such as heart attacks and strokes, and chronic conditions like atherosclerosis and heart failure [4,5,6]. Various mechanisms have been suggested to explain how inhaled particles contribute to cardiovascular disease [4,5,6]. These include the induction of oxidative stress and inflammation, disruptions in autonomic and neuroendocrine regulation, and the direct passage of inhaled particles into the bloodstream [4,5,6].

The harmful health effects of particulate air pollution are evident even at PM2.5 concentrations below current WHO regulatory limits [45,46]. Notably, more than 95% of the global population resides in areas where the WHO’s recommended annual air quality standard for PM2.5 is exceeded [45,46]. Therefore, in addition to reconsidering the current daily PM2.5 standards, it is crucial to implement further strategies aimed at mitigating the harmful effects of particulate air pollution. One promising approach is the use of safe phytochemicals with antioxidant and anti-inflammatory properties [8].

The majority of epidemiological research examining the impact of air pollution on hemostasis has predominantly focused on short-term exposures, typically those lasting less than 7 days [47]. The DEP dose employed in this study aligns with those used in experimental animal models investigating the toxicity of inhaled particulate air pollution and mirrors exposure levels observed in humans living in heavily polluted urban environments [42,48]. Our findings demonstrate that acute lung exposure to DEPs enhanced thrombogenicity in pial arterioles and venules in vivo, increased platelet aggregation in whole blood ex vivo and induced shortening of PT and aPTT in vitro. These findings indicate that DEPs induced hypercoagulability state. Our data align with prior experimental and clinical investigations reporting that inhaled particulate air pollution induces prothrombotic state by enhancing platelet activation, von Willebrand function and the clotting cascade [6,47,49,50,51,52]. While nerolidol has demonstrated some cardiovascular protective effects [13], such as improving endothelial function and reducing oxidative stress, its direct impact on thrombosis is less well-documented. Hence, the present data show for the first time, that nerolidol treatment prevented the prothrombotic effects induced by DEPs in vivo, the platelet aggregation ex vivo and averted the shortening of the PT and aPTT. Recent reports have demonstrated that treatment with other types of phytochemicals such as carnosol, a bioactive phenolic diterpene found in rosemary herb, and nootkatone, a sesquiterpenoid found in grapefruit, alleviates the thrombogenicity induced by lung exposure to DEPs in mice [11,37].

Moreover, our findings demonstrate that i.t. instillation of DEPs significantly increased plasma concentrations of fibrinogen, a critical coagulation protein linked with an augmented risk of coronary events, alongside an increase in PAI-1 and a reduction in tPA levels. In the fibrinolytic process, tPA activates plasminogen to generate plasmin, which breaks down fibrin clots, while PAI-1 inhibits tPA, preventing clot dissolution [47]. The observed decrease in tPA and rise in PAI-1 suggest impaired fibrinolysis, favoring a prothrombotic state. Previous experimental and clinical studies have shown that inhaled particulate air pollution hinders fibrinolysis by increasing PAI-1 and reducing tPA mRNA expression, leading to diminished fibrinolytic activity and fostering a prothrombotic condition [47,53,54]. Our data demonstrate that nerolidol effectively inhibited the DEP-induced increase in plasma concentrations of both fibrinogen and PAI-1 levels, as well as the reduction in tPA. This novel finding, previously undocumented, underscores the significant antithrombotic properties of nerolidol.

It is well established that inhaled pollutants induce lung inflammation, leading to the release of inflammatory cytokines and prooxidants into the systemic circulation [8]. Previous studies have highlighted the pivotal role of systemic inflammation and oxidative stress as key mediators in the cardiovascular effects of particulate air pollution, contributing to diseases and conditions such as atherosclerosis, acute coronary syndrome, stroke, and venous thromboembolism [7,47,51]. Besides preventing the prothrombotic events induced by the pulmonary exposure to DEPs, nerolidol has significantly averted the increase in the plasma concentrations of markers of inflammation (CRP and IL-1β) and oxidative stress (ROS, catalase activity, and NO). Our current findings corroborate previous studies which showed that nerolidol exerts cardioprotective, neuroprotective, and antidiabetic activities through the modulation of oxidative stress and inflammation [13,55,56,57].

Acute exposure to ambient PM2.5 pollution may lead to a substantial increase in key markers of endothelial function, such as ICAM-1, VCAM-1, and selectins indicating potential endothelial dysfunction as a result of exposure to ambient air pollution [58]. The latter can exist in a soluble form in the plasma and are considered as dependable biomarkers of inflammation and endothelial dysfunction [58]. Our data show that exposure to DEPs increased the concentrations of ICAM-1, VCAM-1, E-selectin and P-selectin in the plasma, and that nerolidol administration effectively prevented the rise in these markers of endothelial dysfunction. A recent study has reported that exposure to DEPs in mice caused an increase in the concentrations of ICAM-1, VCAM-1, E-selectin, and P-selectin in aortic tissue, and that the treatment with carnosol abolished this effect [37].

During inflammation, tissue hypoxia arises due to increased oxygen consumption required for the production of inflammatory enzymes and cytokines by both resident and infiltrating cells. Additionally, hypoxia can result from a reduced oxygen supply caused by thrombotic events that obstruct blood flow [59]. A key regulator of cellular adaptation to low oxygen levels is hypoxia-inducible factor (HIF)-1, a protein complex that orchestrates the body’s response to hypoxia [60]. Upon activation, HIF-1 stabilizes, triggering a transcriptional response that upregulates survival factors, including metabolic enzymes, angiogenic factors, and vasoactive substances, to enhance oxygen supply and metabolic activity [59]. Furthermore, oxidative stress has been shown to enhance the expression of HIF-1α. These findings highlight the connection between thrombosis, inflammation, hypoxia, and oxidative stress [61]. In the present study, the exposure to DEPs induced a significant increase in the concentration of HIF-1α in the plasma. This effect could be ascribed to the inflammation, oxidative stress, and hypercoagulability state induced by DEP exposure. Moreover, we have also demonstrated that the treatment with the anti-inflammatory and antioxidant agent nerolidol has potently prevented this effect. A recent study has shown that PM2.5 exposure upregulates HIF-1α in mouse myocardium, leading to myocardial injury and hypertrophy, as well as in an in vitro hypoxia-ischemia model using H9c2 cells [62]. Our data corroborate the findings of a recent study that reported that the treatment with tussilagone, a natural anti-inflammatory compound isolated from the flower buds of *Tussilago farfara*, alleviated lung injury induced by PM2.5 exposure by restoring the increase in HIF-1α and nuclear factor κB in mice [63].

Galectin-3, a β-galactosidase-binding lectin, is key in regulating inflammation. Elevated galectin-3 levels are linked to various cardiovascular disorders [64]. It is primarily produced by immune cells and cardiomyocytes, then secreted into circulation. It can be abnormally overexpressed in conditions such as inflammation, atherosclerosis, and cardiac remodeling [65,66]. Elevated levels of galectin-3 have been detected in the plasma of coronary artery disease patients, where it enhances platelet activation and promotes thrombus formation [67]. Additionally, studies have shown that galectin-3 exhibits prothrombotic and proinflammatory properties in the context of experimental venous thrombosis [68].

Currently, the exposure to DEP induced an increase in the concentration of galectin-3 in the plasma and the treatment with nerolidol has averted this effect. Such an effect has not been reported before. It has been demonstrated that inhaled tobacco smoke increases the expression of galectin-3 in the aortic tissue of mice in vivo, and inhibiting galectin-3 expression in vitro has been reported to decrease cigarette smoke extract-induced autophagy and endothelial progenitor cell dysfunction [69,70]. Moreover, it has also been shown that inhalation of ultrafine carbon particles in mice induces the upregulation of galectin-3 and NGAL expression in alveolar macrophages [71]. Recent studies have shown elevated NGAL plasma activity in coronary artery disease, myocardial infarction, atherosclerosis, and heart failure [72,73]. It has also been demonstrated that NGAL activity exaggerates endothelial cell injury by increasing oxidative stress and inflammation in both normoxic and hypoxic conditions [74]. The expression of NGAL protein by macrophages, smooth muscle cells, and endothelial cells in human carotid atherosclerotic tissue suggests its involvement in establishing the local and systemic proinflammatory environment characteristic of atherosclerosis [75].

In an adult rat model study, NGAL activity in urine was augmented following the exposure to PM2.5 [76]. Additionally, a significant increase in NGAL activity in the plasma of mice exposed to DEPs was observed, and the nerolidol treatment led to the inhibition of this effect. NGAL activity is an acute phase protein which was initially described as a biomarker for acute and chronic kidney injury [77]. Recent studies have shown elevated NGAL plasma activity in coronary artery disease, myocardial infarction, atherosclerosis, and heart failure [72,73]. It has also been demonstrated that NGAL activity exaggerates endothelial cell injury by increasing oxidative stress and inflammation in both normoxic and hypoxic conditions [74].

It is well-established that inflammation and oxidative stress not only cause oxidative DNA damage but also impair DNA repair mechanisms [78,79,80,81]. This impairment can initiate apoptosis, with caspases serving as key mediators in the process. Among them, caspase-3 plays a crucial role in executing apoptosis [78,79]. Our findings reveal an increase in plasma levels of the oxidative DNA damage marker 8-OH-dG and the apoptosis marker cleaved caspase-3 following DEP exposure. Notably, treatment with nerolidol effectively prevented these effects. Our data align with recent studies demonstrating that nerolidol attenuates inflammation, oxidative stress, and apoptosis in doxorubicin-induced acute cardiotoxicity in rats [56].

This study has some limitations. Here, we have assessed the effect of DEPs as such. Given the fact that DEPs are made up of a carbonaceous core with other chemicals adsorbed onto them, such as nitrates, sulfates, polycyclic aromatic hydrocarbons, and trace heavy metals, it remains to be established which constituent(s) of DEPs is responsible for the observed effect [82,83]. The biochemical analysis in other organs such as the lungs, heart, and kidneys were not performed in this study, and will be assessed in a separate study which will involve different techniques such as Western blot, immunohistochemistry, and flow cytometry. Also, exploring the polarization of lymphocytes in response to DEP exposure and the possible protective effect of nerolidol on other forms of air pollution remain to be explored. Furthermore, conducting additional studies on the pharmacokinetic profile of nerolidol—such as investigating peak plasma concentrations and half-life—could offer a clear understanding of how nerolidol exerts its mitigating effects at the dose used presently.

In conclusion, our data demonstrate that nerolidol exerted ameliorative actions against DEP-induced peripheral thrombosis, endothelial dysfunction, systemic inflammation, oxidative stress, DNA damage, and apoptosis. Pending further research, nerolidol could be regarded as a promising agent with the potential to alleviate the harmful effects experienced by individuals residing in urban areas with elevated levels of particulate air pollution.

## Data Availability

Data underlying the findings of this study can be obtained from the corresponding author, Abderrahim Nemmar, upon reasonable request.
